# Thermally Driven Formation of Multiphase, Mixed-Dimensional Architectures from TaSe_3_ Nanoribbons

**DOI:** 10.1021/acsnano.5c13312

**Published:** 2025-10-20

**Authors:** Casey F. Rowe, Eric V. Formo, Jordan A. Hachtel, Tina T. Salguero

**Affiliations:** † Department of Chemistry, 1355 University of Georgia, Athens, Georgia 30605, United States; ‡ Georgia Electron Microscopy, University of Georgia, Athens, Georgia 30605, United States; § Center for Nanophase Materials Sciences, 6146 Oak Ridge National Laboratory, Oak Ridge, Tennessee 37830, United States

**Keywords:** in situ STEM, core−shell, quantum material, transition metal chalcogenide, one-dimensional, mixed-dimensional, heterostructure

## Abstract

Tantalum–selenium compounds, particularly TaSe_2_ and TaSe_3_, are promising materials for electronics and quantum technologies due to their charge density wave and topological properties, and they are also candidates for energy storage and electrocatalysis applications. In this study, we investigate the thermally driven structural evolution of TaSe_3_ nanoribbons using *in situ* scanning transmission electron microscopy (STEM). Low-kV STEM experiments reveal a complex nanoscale transformation pathway in which TaSe_3_ nanoribbons convert into multiphase, mixed-dimensional (0D–1D) tantalum–selenium architectures. Aberration-corrected STEM enables direct visualization of the underlying atomic rearrangements, while electron energy loss spectroscopy and DFT calculations corroborate the identity and stability of the product phases. Our results uncover a detailed mechanism: selenium loss from TaSe_3_ nanoribbons initiates surface conversion to TaSe_2_, which, as temperature increases, progressively continues into the nanoribbon interior. Thicker regions of TaSe_2_ delaminate and detach from the core material, forming a porous TaSe_2_ shell. At 1200 °C, the core restructures into discrete ∼20 nm Ta-self-intercalated TaSe_2_ nanoparticles. This core–shell transformation, driven by nanoscale confinement effects, differs markedly from the bulk decomposition pathway of TaSe_3_ and highlights the impact of modulating selenium loss, tantalum intercalation, and the stability of intermediate structures through confinement effects. The resulting 0D–1D heterostructure of Ta-rich nanoparticles encapsulated within porous TaSe_2_ tubes represents surprising and emergent complexity in a binary system. These mechanistic insights demonstrate how the controlled thermolysis of a readily accessible metal trichalcogenide precursor can yield complex, low-dimensional chalcogenide architectures.

## Graphical abstract

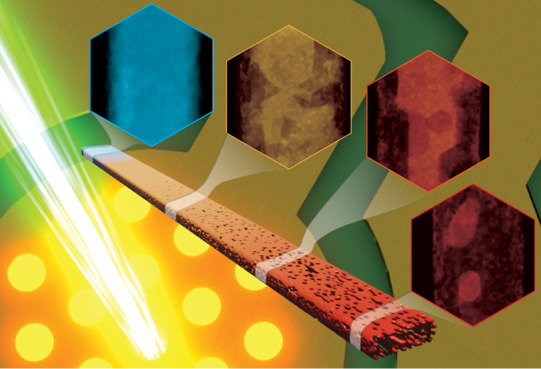

## Introduction

One-dimensional (1D) transition metal chalcogenides are attracting growing attention for their structural and electronic properties that offer promising avenues for next-generation electronic, optoelectronic, and quantum devices.^1–4^ Among their distinguishing features is the possibility to exfoliate these materials to near-atomic single chain thicknesses, owing to weak van der Waals (vdW) interactions between chain subunits.^5–8^ Within this class, MX_3_-type compounds (M = Ti, Zr, Hf, Nb, Ta; X = S, Se, Te) exhibit a range of interesting phenomena including charge density waves, superconductivity, and topologically nontrivial states.^2,9^

TaSe_3_ is a particularly intriguing example. It crystallizes in a monoclinic MX_3_-type structure consisting of bilayers of type-I and type-II trigonal prismatic TaSe_6_ chains and is considered a quasi-1D material.^10,11^ Since the discovery of its low-temperature superconductivity (T_s_ ≈ 2.1 K) in 1977, TaSe_3_ has been studied for both its charge-density-wave behavior and its predicted topological surface states.^11–15^ In addition, TaSe_3_ nanoribbons have shown strong potential for nanoscale device applications, including high-performance interconnects and electromagnetic shielding.^16–19^

Despite these advances, little is known about the thermal stability of TaSe_3_ nanoribbons, particularly under elevated temperatures relevant to device integration (RT–500 °C) or to accessing solid state chemistry (>500 °C).^20^ Although prior studies showed that TaSe_3_ decomposes to TaSe_2_ + Se upon heating to 500 °C under thermogravimetric analysis (TGA) conditions, this pathway may not translate exactly to samples experiencing nanoscale confinement.^21^ In our prior *in situ* electron microscopy work on NbS_3_–IV nanoribbons, for example, we elucidated a topotactic pathway leading to nanostructured NbS_2_.^22^ These findings raise a key question: does TaSe_3_ follow a similar topotactic pathway upon thermolysis, or do its unique structure and chemistry give rise to unexpected behaviors?

In this study, we investigate the high-temperature transformation of TaSe_3_ nanoribbons using *in situ* electron microscopy. Using low-kV scanning transmission electron microscopy (STEM), aberration-corrected STEM, and electron energy loss spectroscopy (EELS), we capture a surprising sequence of structural changes. Rather than directly forming TaSe_2_, TaSe_3_ nanoribbons heated up to 1200 °C evolve spontaneously into a mixed-dimensional, multiphase architecture: comprehensive imaging and analysis reveal several intermediate states leading to a porous, tubular TaSe_2_ shell encapsulating discrete ∼20 nm Ta-rich, self-intercalated TaSe_2_ nanoparticles. We propose that this transformation is significantly influenced by nanoscale confinement to deviate from conventional bulk decomposition pathways.

The appearance of nanostructured TaSe_2_ in the product is especially notable given the growing technological relevance of TaSe_2_ in its own right. Depending on its polytype (e.g., 1*T* or 2*H*), TaSe_2_ exhibits charge density wave transitions, low-temperature superconductivity, and rich optical and electronic behavior.^23–26^ Its two-dimensional nature has made it an attractive platform for vdW heterostructures, broadband photodetectors, saturable absorbers, and hydrogen evolution catalysis.^27–30^ The ability to generate porous, nanoscale TaSe_2_ architectures through the thermolysis of TaSe_3_ nanoribbons thus presents a novel route for fabricating functional forms of this material.

Together, these findings reveal a previously unreported transformation pathway for an MX_3_-type compound and demonstrate how such a precursor can be used to generate emergent 0D–1D heterostructures. This thermally driven conversion offers new opportunities for controlling phase, morphology, and composition in tantalum chalcogenides, and suggests broader strategies for accessing complex metal–chalcogenide nanostructures through controlled decomposition processes.

## Results and Discussion

TaSe_3_ crystals were prepared using a melt-flux method and characterized by powder X-ray diffraction and scanning electron microscopy-energy dispersive X-ray spectroscopy (SEM-EDS) to confirm structure and composition (Figure S1D,E).^31^ The thermal behavior of bulk TaSe_3_ was reassessed by thermogravimetric analysis (TGA), demonstrating an overall weight loss of 59.1% between 250 and 1000 °C (Figure S1F), corresponding to nearly complete loss of Se. The majority of this decomposition occurs between 300 and 521 °C (49.6%), and there is an additional small yet distinct weight loss at 848–905 °C (1.9%).^32,33^

TaSe_3_ crystals were exfoliated through probe sonication in ethanol to produce a dispersion of TaSe_3_ nanoribbons. This dispersion was applied onto the microelectromechanical system (MEMS) chips of each *in situ* heating holder. The criteria for selecting particular nanoribbons to monitor during *in situ* experiments included (1) deposition into those MEMS chip wells located over vacuum, and (2) nanoribbon widths of 60–100 nm to maximize the image quality from SE, BF, and HAADF detectors.

### Low kV *In Situ* STEM Experiments

Low kV *in situ* STEM analysis provided an initial overview of thermally induced changes to the structure of TaSe_3_ nanoribbons (Figures 1 and S2). By concurrently collecting data with a combination of secondary electron (SE), bright field (BF), and high angle annular dark field (HAADF) detectors, we identified changes to the nanoribbon’s surface morphology in addition to internal changes of its material distribution and composition. The TaSe_3_ nanoribbon shown in Figure 1 begins with a diameter of 135.3 ± 1.3 nm at RT; it has an overall smooth, uniform appearance in SE mode (Figure 1A), and the BF and HAADF detectors indicate an even distribution of internal material (Figure 1B,C). At up to 200 °C, little to no change can be detected by any imaging mode. At 300 °C the organic overlayer, likely including adsorbed ethanol, dissipates due to elevated temperature, yielding a considerably clearer view of surface structure and a reduced nanoribbon diameter of 127.1 ± 1.5 nm from SE imaging (change of −6.1% from RT) (Figure S3).

**Figure 1:**
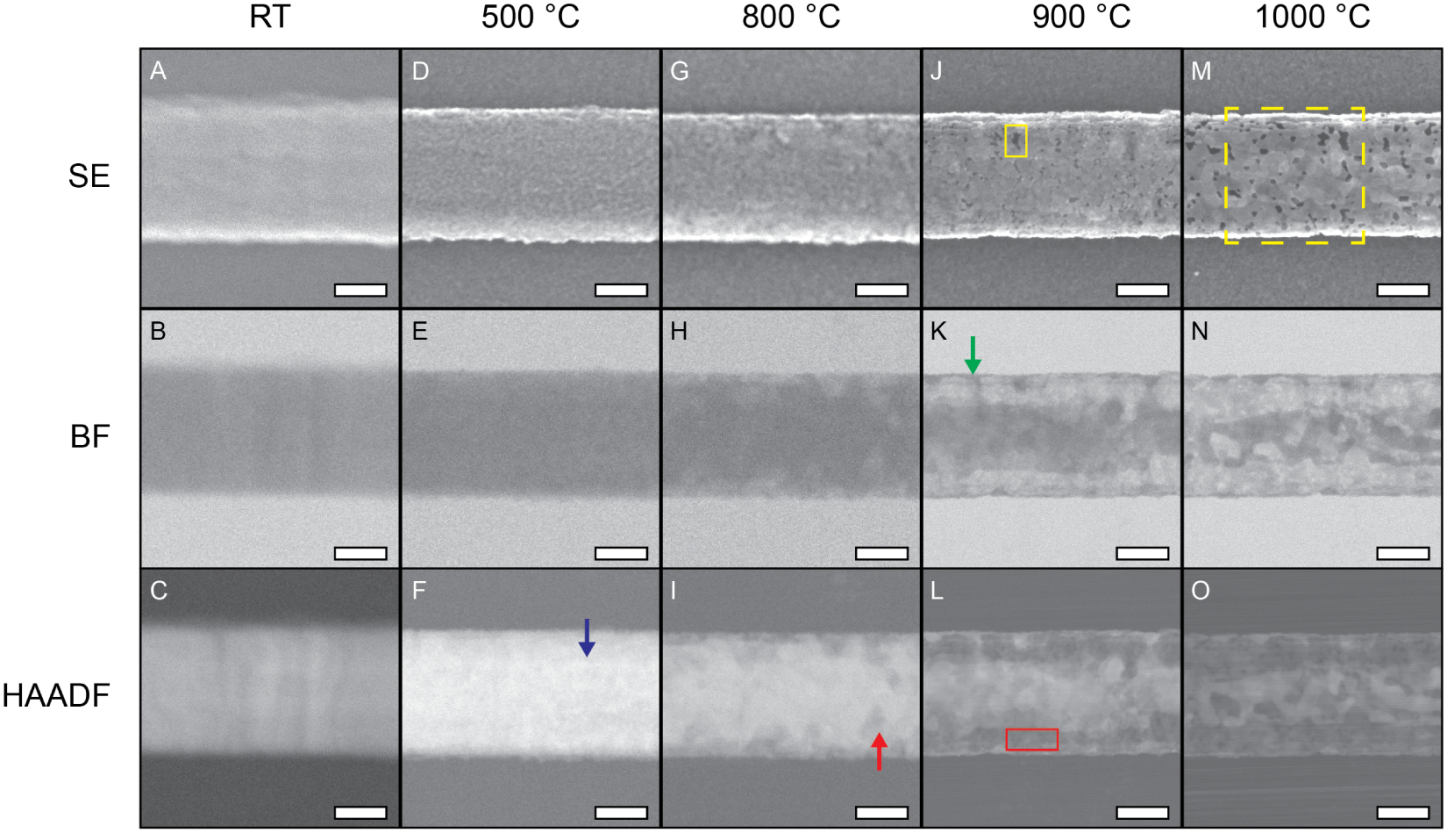
Data from key temperature steps during the low kV *in situ* STEM thermolysis of a TaSe_3_ nanoribbon, collected using the secondary electron (SE), bright field (BF), and high-angle annular dark field (HAADF) detectors of a Hitachi SU9000EA microscope. The organic overlayer at RT (A–C) dissipates after heating, clearly revealing the nanoribbon morphology and internal material distribution at 500 °C (D–F). By 800 °C, the nanoribbon begins to show signs of transformations, primarily at peripheral sites (G–I). At 900 °C, the surface morphology is consistent with a porous shell (J), and BF and HAADF views expose the core–shell structure (K,L). These features become more pronounced at 1000 °C (M–O); the yellow dashed box in panel (M) corresponds to the area shown in Figure S4. Scale bars = 50 nm.

At 500 °C, the onset of pitting becomes visible across the nanoribbon in the HAADF data, which also reveals the emergence of contrast variations between regions of differing material density (Figure 1F, blue arrow), which will continue to develop into a core–shell structure at higher temperatures. As the sample reaches 800 °C, more pronounced changes become apparent in all imaging modes. The surface morphology shows rough, textured patches that are most clearly visible along the periphery (Figure 1G). These morphological developments are corroborated by the BF and HAADF images (Figure 1H,I), which reveal polyhedral incursions extending from the periphery inward. These features appear to consume material progressively as they propagate toward the core, suggesting mass loss associated with Se volatilization. Similar behavior has been reported in related systems involving chalcogen loss upon heating.^22,34–37^ Additionally, a few polyhedral divots appear in the sample’s interior that are spatially isolated from those extending from the periphery (Figure 1I, red arrow). In areas experiencing material loss, a thin residual layer remains where the TaSe_3_ nanoribbon exterior once existed.

At 900 °C the atomic density near the nanoribbon’s periphery decreases considerably in comparison to an emerging core of the nanoribbon, which is reflected in increased relative intensity of the BF signal and the decreased relative intensity of the HAADF signal. It is important to note that BF imaging, which relies on phase contrast, can produce misleading contrast effects. However, HAADF imaging relies on *Z*-contrast, or atomic number contrast, meaning the resulting contrast can be interpreted as a difference in the effective projection-integrated atomic number. Therefore, the reduced HAADF contrast demonstrates a reduction in the total material at the nanoribbon periphery (with respect to the core). The result is a pitted, porous surface (Figure 1J, yellow box). These pores range in area between 1.4–38.9 nm^2^ with a mean area of 8.1 nm^2^. Concurrently, the internal material undergoes substantial changes as the development of a unique core–shell nanostructure is revealed by the BF and HAADF images (Figure 1K,L). In the region that previously formed the nanoribbon periphery, a thin strip of porous material (Figure 1K, green arrow) constitutes a “shell” that maintains the overall shape and width of the nanoribbon. The former central volume of the nanoribbon shows an approximately cylindrical core with a density greater than the shell based on contrast differences in BF and HAADF modes. To starkly differentiate the core–shell features, we highlight the void space formed between the core and shell (Figure 1L, red rectangle) that points to a hollowing process. Nanoribbon measurements based on the BF images show a width reduction from 121.5 ± 2.2 nm at RT to 118.3 ± 1.5 nm at 900 °C (change of −2.6%), suggesting that the shell is robust and serves to support the overall evolving architecture (Figure S3).

Remarkably, the core–shell structure persists at 1000 °C, despite a cumulative 6 h of continuous heating across the preceding temperature steps. Surface pores visible in SE images have expanded significantly (Figure 1M), with pore areas ranging from 1.7 to 180.0 nm^2^ (mean of 28.2 nm^2^). BF and HAADF images confirm the continued presence of the core–shell structure (Figure 1N,O), although the core appears to have a considerably diminished density. These observations suggest that the shell initially functions as a protective barrier to preserve the core’s structure. With continued heating, however, the shell becomes increasingly porous and the core restructures into highly irregular, though centralized, particle agglomerates (Figure S4).

Other studies involving the *ex situ* annealing of NbSe_3_ and TaS_3_ nanoribbons have postulated core–shell formation based on transmission electron microscopy imaging, electron diffraction, and X-ray photoelectron spectroscopy data. In these cases, the exact structures and compositions of the resulting core and shell were not identified.^34,37^ In comparison, here our low kV *in situ* STEM thermolysis results provide direct evidence of a core–shell structure first appearing by 500 °C and progressively developing into a well-defined architecture characterized by a central, dense core, surrounding void space, and a thin and porous tubular shell. In our prior *in situ* STEM thermolysis study of NbS_3_–IV, we observed a distinctly different transformation pathway: a topotactic mechanism yields NbS_2_ grains aligned with the nanoribbon axis, accompanied by the formation of polyhedral divots and plateaus, but without the development of a surrounding shell.^22^ Thus, the core–shell transformation of TaSe_3_ described here represents a previously unreported pathway for MX_3_-type TMCs.

### Aberration-Corrected *In Situ* STEM Annealing Experiment

*In situ* heating experiments using aberration-corrected STEM yielded key insights into the chemical and structural changes underlying this unique core–shell transformation. Atomic-resolution imaging provided direct insight into the crystal structure during key steps of the reaction. To systematically examine this process, we performed three sequential annealing steps on a single nanoribbon, with RT imaging after each to examine the atomistic structure evolution. This data set is summarized in Figure 2.

**Figure 2:**
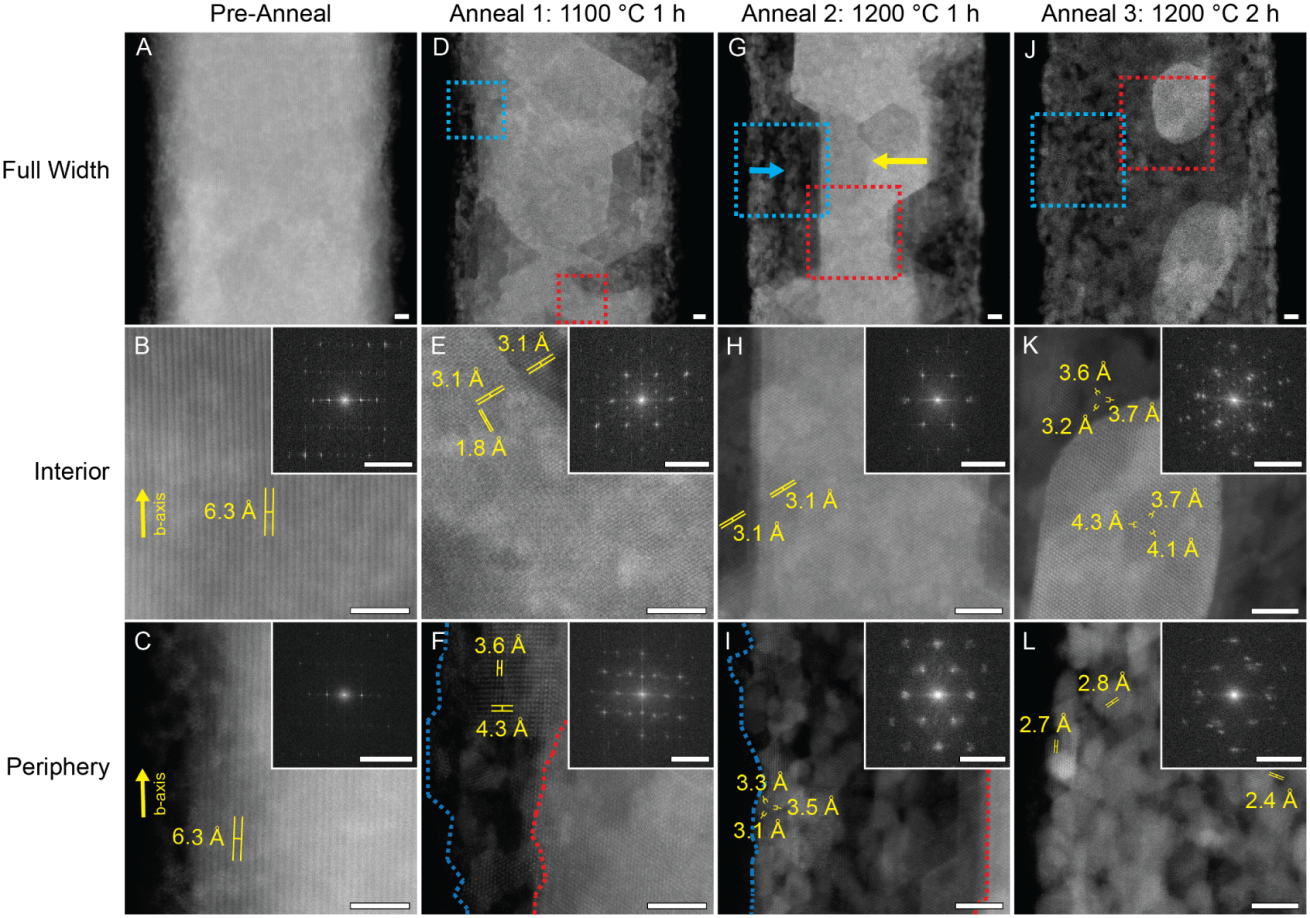
TaSe_3_ nanoribbon imaged using a Nion UltraSTEM 100 microscope during the annealing experiment. Views feature the full-width nanoribbon overview, interior, and periphery. The nanoribbon prior to heating (A–C) shows spacings and FFT patterns consistent with TaSe_3_. After initial annealing treatment at 1100 °C (D–F), polyhedral incursions into the core material appear alongside the development of a porous shell at the periphery. Further annealing at 1200 °C (G–I) results in a clearly established core (yellow arrow) surrounded by a shell (blue arrow) with a mottled appearance. Further heat treatment at 1200 °C (J–L) results in core collapse into discrete nanoparticles. Micrograph scale bars = 5 nm. FFT inset scale bars = 0.5 Å^–1^.

Prior to annealing, the TaSe_3_ nanoribbon exhibits an even distribution of material (Figure 2A). Decreases in *Z*-contrast toward the exterior can be attributed to thinning of the sample at the periphery due to rounding. Upon closer inspection, vertical striations parallel to the nanoribbon axis with atomic spacing measurements of 6.3 Å are consistent with the TaSe_3_ bilayers aligned along the *b*-axis; the corresponding fast Fourier transform (FFT) of the full image further validates this orientation (Figure 2B,C).^10^

After annealing at 1100 °C for 1 h (Figure 2D), the sample reveals pronounced structural and morphological changes. Most notably, polyhedral incursions become visible, accompanied by variations in image contrast, propagating inward from the periphery. At the same time, material at the periphery begins to adopt a mottled, irregular, and porous appearance consistent with observations from the low kV STEM experiment. Figure 2E,F corresponds to higher magnification views of the interior and periphery, respectively, as indicated by the red and blue dashed boxes in Figure 2D. In the interior region (Figure 2E), the atomic rows no longer appear oriented parallel to the *b*-axis, and the lattice spacings are significantly reduced compared to those of TaSe_3_. The measured spacings of 1.8 Å and 3.1 Å are consistent with theoretical values for adjacent Ta atoms in 2*H*-TaSe_2_ viewed along the <101> direction (Figure 2E).^38^ The corresponding FFT shows an overarching change from the original tetragonal symmetry (Figure 2B inset) to a dominant hexagonal pattern (Figure 2E inset), further confirming the formation of TaSe_2_. A secondary hexagonal FFT pattern rotated by 30° indicates the presence of multiple domain orientations. The darker regions near the top and lower left of the interior can be attributed to selenium depletion from the starting TaSe_3_ lattice.

Figure 2F shows the structural changes occurring at the sample’s periphery. A growing void space develops between the inner core (red dashed line) and the surrounding shell (blue dashed line), with an average separation of 7.5 ± 1.4 nm. Additionally, a particle seen near the periphery exhibits a tetragonal lattice with measured spacings of 4.3 Å and 3.6 Å. Although 2*H*-TaSe_2_ viewed along the <100> direction can appear tetragonal, the observed lattice constants are smaller than expected, suggesting a structural change.

Determining the specific TaSe_2_ polytype(s) formed during this transformation is challenging due to the substantial similarity in atomic spacings across polytypes. All TaSe_2_ polytypes possess in-plane Ta–Ta distances of ∼3.0 Å when viewed along the <100> direction, and out-of-plane spacings of ∼3.4 to 3.5 Å along the [001] direction. Ta–Ta spacings measured along <101> directions offer small distinctions among polytypes (Table S1). Compounding this challenge is the effect of nanoscale confinement on polytype stability, which likely deviates from bulk behavior. In bulk, 2*H*-TaSe_2_ is thermodynamically favored at RT, whereas 1*T*-TaSe_2_ is considered metastable and has been reported to form on the surface of TaSe_3_ nanofibers at ∼700 °C.^35^ On the other hand, Wu et al. reported the formation of 3*R*-TaSe_2_ nanobelt quasi-arrays following the pyrolysis of TaSe_3_ nanobelts at 550 °C.^36^ These contrasting outcomes underscore the sensitivity of TaSe_2_ polymorphism to subtle differences in local environment and formation conditions. Our data do not allow us to assign a definitive polytype to the evolving core–shell structures observed in this study.

A second annealing step at 1200 °C for 1 h accelerated the transformation process. As seen in Figure 2G, substantial structural changes include the development of a well-defined, faceted core (yellow arrow), formation of a distinct porous shell (blue arrow) closer to the center, and further expansion of the void space between core and shell, likely caused by continued selenium loss during this annealing step. Higher-resolution imaging of the interior region (Figure 2H) revealed that both the core and the adjacent interface zone have lattice spacings of 3.1 Å, consistent with TaSe_2_ and indicating that variations in image contrast are due to density or orientation differences rather than different phases.

At the periphery (Figure 2I), the shell is composed of grains in multiple crystallographic orientations. The dominant structure within this region adopt a hexagonal arrangement with interatomic spacings of 3.1 Å, 3.3 Å, and 3.5 Å—values corresponding to Ta–Ta distances in TaSe_2_ viewed along the [001] direction, with minor deviations attributable to slight sample tilting.^38^ Other regions of the shell show variations in both contrast and focus, indicating a fused, polycrystalline shell structure with significant 3D topography; this interpretation was corroborated by low- and high-defocus imaging (Figure S5). The corresponding FFTs for both core and periphery sites (Figure 2H,I insets) confirm that hexagonal symmetry dominates these patterns. Additionally, the core’s outer boundary (red dashed line, Figure 2I) has moved further inward, expanding the void between core and shell to an average distance of 24.5 ± 1.1 nm. Figure S6 provides additional data illustrating the characteristic features of the three distinct regions that formed consistently during this thermolysis process: a central crystalline core, a surrounding porous shell, and an intermediate region at the core–shell interface.

Following a third annealing step at 1200 °C for an additional 2 h, the central core undergoes collapse, fragmenting into discrete nanoparticles ranging from 13 to 22 nm in width (Figures 2J and S7). These particles are considerably brighter in contrast relative to the shell material and lack the well-defined polyhedral edges observed previously. The particles appear randomly dispersed within the tubular shell, which includes increased void spaces between fused sections and further overall structural coarsening.

Closer inspection of these core particles reveals interesting differences in atomic structure. Although both the core particles and shell display hexagonal symmetry, the particles exhibit distinctly larger lattice spacings of 4.3 Å, 4.1 Å, and 3.7 Å (Figure 2K). In contrast, the shell material maintains the more typical TaSe_2_ spacings of 3.7 Å, 3.6 Å, and 3.2 Å (Figure 2L). Optimized defocus imaging confirmed these expanded spacings in the core particles, which do not match any known Ta–Se or elemental tantalum phases (Table S1). Furthermore, FFT analysis shows a more complex pattern composed of a rotated hexagonal signal associated with the shell superimposed with a new set of reflections corresponding to the core phase (similar to features seen earlier during anneal 1). These results suggest the formation of a unique or metastable Ta–Se phase stabilized by nanoconfinement.

In the final state, the shell becomes the dominant structural component of the sample. It consists of crystalline grains ranging from 1.9 to 4.7 nm in diameter (Figure 2L), with a range of lattice spacings corresponding to various crystallographic orientations. The elongated spot pattern of the FFT is consistent with this polycrystalline nature. Notably, despite the extensive internal structural transformation, the overall width of the sample remains nearly unchanged compared to the original TaSe_3_ nanoribbon; starting from a preanneal width of 78.0 ± 1.4 nm, the width after anneal 3 is 77.3 ± 1.1 nm (Figure S8). Such a minimal change is atypical for MX_3_ to MX_2_ conversions, where significant diameter reduction usually accompanies chalcogen loss.^22^ In addition, we note that cooling did not reverse or further alter any products and thus all observed transformations were irreversible.

### Core Particle Analysis

Aberration-corrected STEM enables the deeper exploration of chemical transformations within materials through electron energy loss spectroscopy (EELS). EEL spectra were acquired from the interior regions of the sample at RT and after each annealing step (Figure 3A,B). At RT, the volume plasmon peak (20.6 eV) can be identified readily, whereas the Ta^4+^/Ta^5+^ mixed valence state of TaSe_3_ produces a broader, less resolved signal corresponding to the Ta–O_2,3_ edge (∼39 eV).^4^ The Se-M_4,5_ edge (55.7 eV) also presents as a minor peak. After the first anneal, changes reflect a shift in tantalum oxidation state toward Ta^4+^ characteristic of TaSe_2_. This transition results in the appearance of distinct Ta–O_2_ (47.3 eV) and Ta–O_3_ (39.0 eV) edge peaks, consistent with previous literature.^39^ The second anneal induces no further changes to the spectrum because the composition remains TaSe_2_. However, the EEL spectrum of a core particle formed during the third anneal reveals new features: three additional peaks at 14.3, 25.3, and 27.2 eV near the volume plasmon peak region. This change is consistent with peak broadening caused by an increase in electron density through, for example, the presence of a metal like Ta. We note that previously reported EEL spectra for the volume plasmon energies of Ta and TaN resemble the core particle spectrum observed here with respect to similar energy losses and peak broadening.^40^ Moreover, the calculated free-electron plasma energy for *bcc* Ta reported by Weaver and coworkers agrees well with our core particle EEL data.^41^ The core particle spectrum also includes features from electron beam interactions with the TaSe_2_ shell.

**Figure 3:**
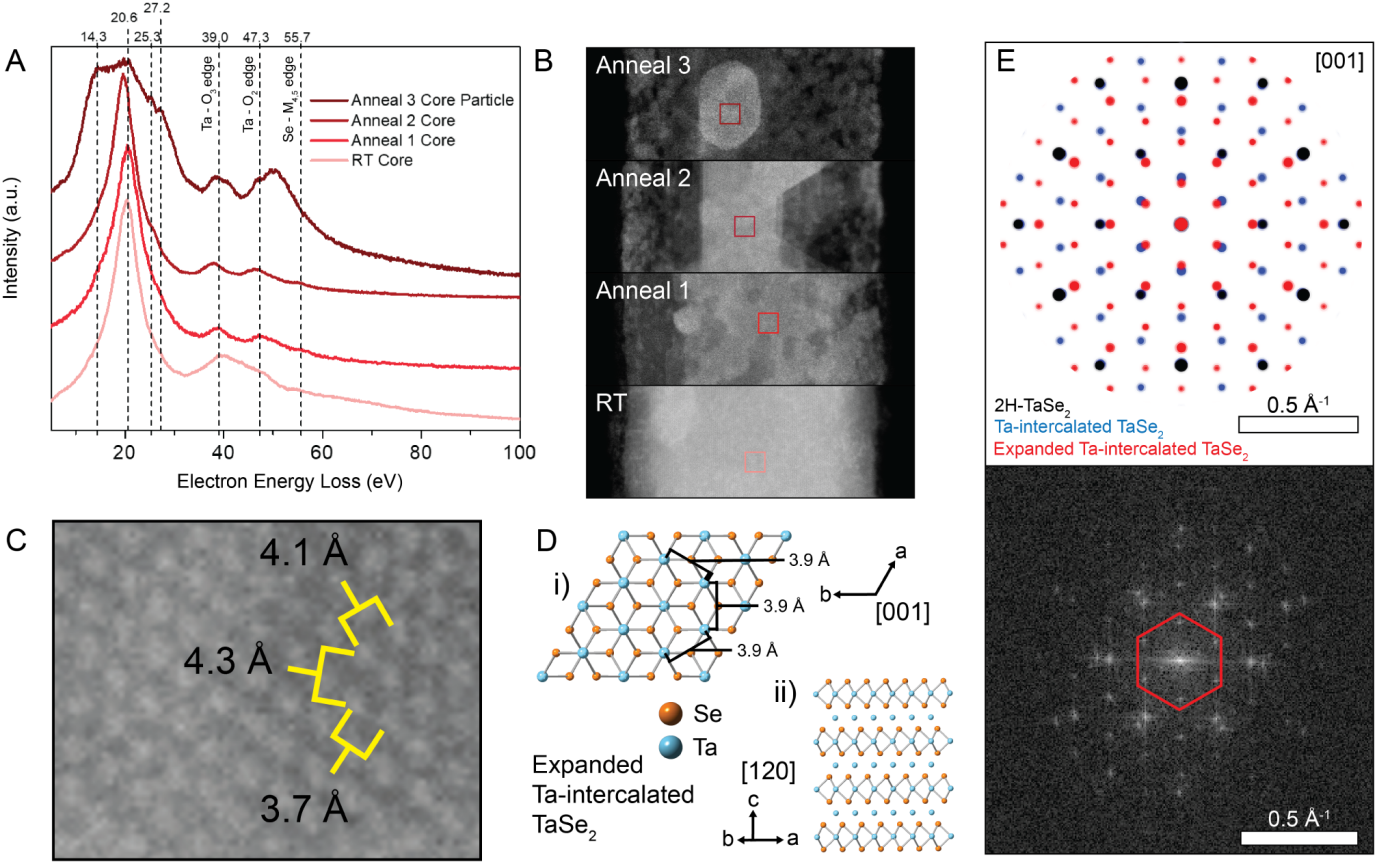
(A) Normalized EELS for interior locations within the nanoribbon at each anneal step. (B) STEM images showing approximate areas of EELS collection at each anneal step (red squares). (C) Atomic-resolution electron micrograph of a core particle formed after the final anneal with measured spacings. (D) Crystal structure based on DFT-optimized hexagonal TaSe_2_ with intercalated Ta, showing (i) calculated spacings similar to observed particle spacings when viewed in the [001] orientation and (ii) Ta atoms intercalated between TaSe_2_ layers when viewed in the [120] direction. (E) Top: simulated electron diffraction patterns for 2*H*-TaSe_2_ (black spots), Ta-intercalated TaSe_2_ (blue spots), and expanded unit cell Ta-intercalated TaSe_2_ (red spots) as viewed down the *c*-axis. Bottom: observed FFT pattern for a representative core particle.

On the other hand, the measured atomic spacings of the core particles are significantly larger than those of Ta, and the corresponding FFT pattern shows smaller *d*-spacings that do not match the lattice parameters of *bcc* Ta. These inconsistencies suggested that the core particles are not simply Ta but a more complex phase, prompting further structural investigations.

A thorough examination of the lattice and FFT pattern of the core particle led us to conclude that its structure closely resembles that of TaSe_2_. Oxidation to cubic TaO was ruled out based on both EDS analysis and a mismatch between the much smaller *d*-spacings of TaO and those observed experimentally (Figures S9 and S10). Comparisons with eight other known Ta–Se and Ta phases yielded no structural matches to the experimental data (Table S1). Furthermore, the broadened EELS plasmon features, combined with the high contrast of the core particle relative to the darker TaSe_2_ shell, suggest an increased Ta:Se ratio. These observations led us to consider the possibility of Ta self-intercalation within the vdW gaps in TaSe_2_, leading to a composition of Ta1+_*x*_Se_2_ (where *x* reflects the amount of intercalated Ta). Self-intercalation of metal cations into 2D MX_2_-type materials is well-established in the literature.^42–44^ For example, Zhao et al. reported the growth of Ta-self-intercalated TaS_2_ and TaSe_2_ via molecular beam epitaxy and chemical vapor deposition, with intercalation concentrations tuned by varying the Ta:X flux ratios.^42^ Their findings indicated that the intercalated Ta atoms preferentially occupy octahedral interlayer sites, resulting in a layered stacking sequence TaX_2_–Ta–TaX_2_–Ta.

To evaluate the plausibility of a Ta-intercalated TaSe_2_ phase, we performed density functional theory (DFT) calculations based on a known V-intercalated Ta_3_VS_6_ structure, substituting V and S atoms with Ta and Se, respectively.^45^ The resulting optimized structure of Ta-self-intercalated TaSe_2_ provided lattice parameters of *a* = *b* = 6.0331 Å, *c* = 13.2285 Å, α = β = 90°, γ = 120°. The calculated formation enthalpy (Δ*H* = −0.69378 eV per atom) lies above the hull energy, suggesting that this structure is metastable (Figure S11).^46,47^ Entropic contributions from experimental conditions above 0 K may shift this phase toward thermodynamic stability. In addition, the simulated spacings in the [001] direction based on this optimized structure still produce atomic spacings similar to unmodified TaSe_2_, which is consistent with previous studies demonstrating that metal cation intercalation into transition metal dichalcogenides impacts only the *c*-axis while preserving the in-plane lattice.^48,49^

To reconcile the discrepancy between simulated and experimental spacings, we modeled an expanded unit cell with lattice parameters *a* = *b* = 6.8 Å and *c* = 17.0 Å. When viewed along the [001] zone axis, this structure exhibits projected spacings of 3.9 Å (Figure 3D), which align more closely with the experimental value for the core particle (Figure 3C). We note that in Figure 3C, the atomic lattice appears slightly distorted due to imaging the core particle slightly off the [001] zone-axis. FFT analysis further supports this interpretation: the inner hexagonal pattern in the experimental FFT (red hexagon) features *d*-spacings of 6.0 Å, which match the simulated diffraction pattern of expanded Ta-intercalated TaSe_2_ (red dots, 5.88 Å). In comparison, the unexpanded intercalated phase has smaller *d*-spacings (blue dots, 5.15 Å), and TaSe_2_ lacks a comparable hexagonal pattern (black dots). This expanded unit cell structure has a calculated formation enthalpy of 3.73783 eV/atom, far above the hull energy, indicating a high degree of instability (Figure S11). However, atomic spacing analysis of the core particle shows as much as 10% variation in interatomic distances (Figure S12), suggesting that Ta may be occupying interstitial sites in addition to interlayer sites, further perturbing the local lattice, Ta–Ta spacings, and unit cell parameters. These factors, combined with elevated thermal conditions, may contribute to phase stabilization under these *in situ* conditions.

This interpretation of the composition and structure of the core particle also appears consistent with observations from the first annealing step at 1100 °C. As described earlier, that FFT pattern includes a rotated internal hexagonal structure (Figure 2E inset) similar to that of the core particle after anneal 3. The atomic spacings of the particle in Figure 2F at the nanoribbon periphery—4.3 Å and 3.6 Å—closely match the simulated Ta–Ta distances (4.3 Å and 3.4 Å) for the expanded lattice Ta-intercalated TaSe_2_ in the <210> directions, which suggests that Ta self-intercalation also may occur at the exterior of the sample. We propose that the formation of void spaces between the core and shell precludes additional widespread intercalation at the exterior even as the core continues to lose Se.

### Aberration-Corrected *In Situ* STEM Temperature Increment Experiment

Figure 4 presents additional atomic resolution aberration-corrected STEM imaging for the *in situ* thermolysis of another TaSe_3_ nanoribbon. The focus of this data collection was capturing the complete transformation sequence by evaluating structural changes as temperature increased in 100 °C steps. At RT, the TaSe_3_ chain structure (viewed down the *a*-axis) is clearly resolved, with interatomic Ta–Ta spacings of 3.6 Å and 2.5 Å in good agreement with expectations (Figure 4A).^10^ By 400 °C, the first sign of structural transformation appears at the nanoribbon periphery in the form of hexagonal domains (Figure 4B, right side). The measured atomic spacings within these domains match those of TaSe_2_ when viewed along the [001] direction. However, the FFT still reveals a dominant TaSe_3_ phase, which is consistent with the continued presence of vertically aligned chains in the untransformed core region (Figure 4B, left side). At 500 °C, the hexagonal regions extend further toward the ribbon interior and the FFT exhibits more pronounced hexagonal symmetry (Figure 4C).

**Figure 4:**
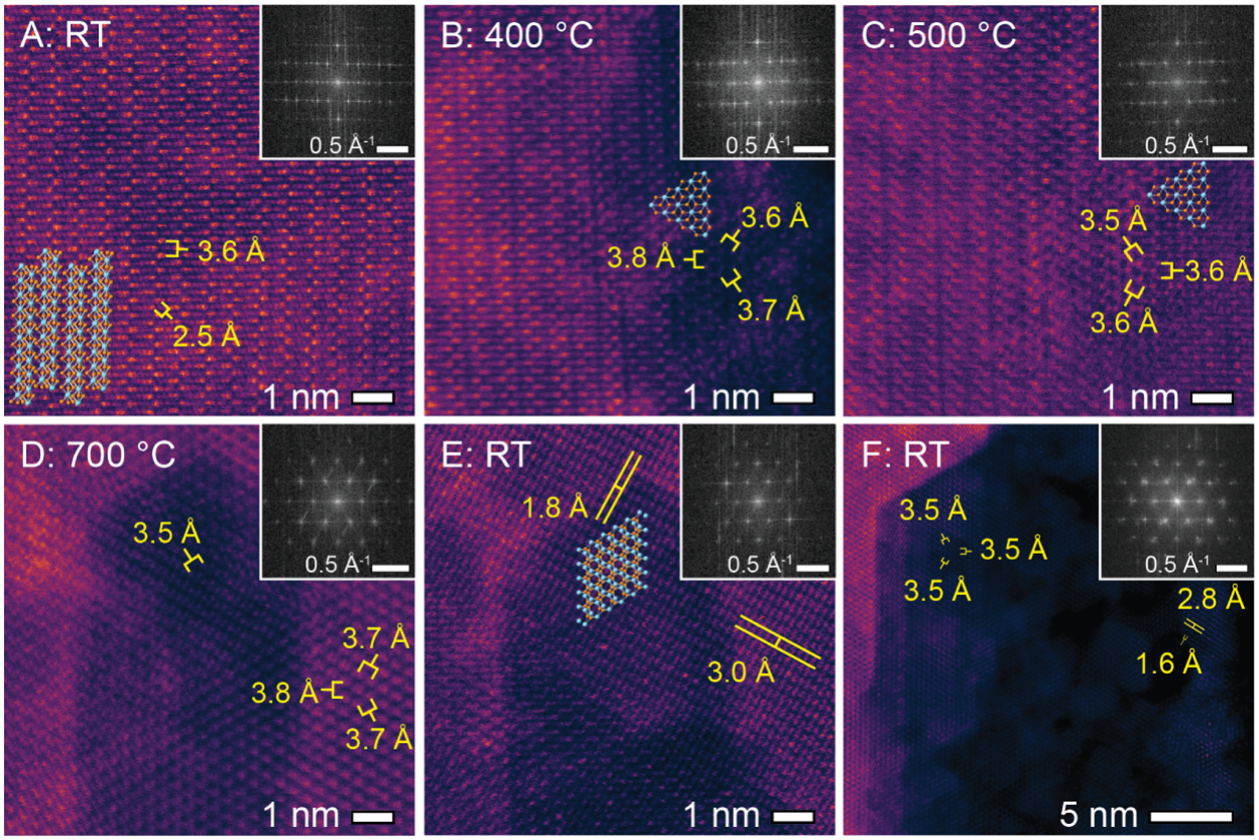
Atomic-resolution images of a TaSe_3_ nanoribbon at various temperature steps with corresponding FFT insets. The RT image of the sample prior to heating (A) exhibits spacings and FFT consistent with TaSe_3_; the crystal structure is overlaid. At 400 °C, hexagonal lattice features begin to appear at the nanoribbon periphery (B), and by 500 °C, similar hexagonal domains appear in the interior (C); overlays of the 2*H*-TaSe_2_ crystal structure are included in panels (B,C). At 700 °C, polyhedral divots with predominantly hexagonal FFT patterns (D) are visible. After reaching 1200 °C and returning to RT, the core retains polyhedral features consistent with 2*H*-TaSe_2_ (E), and the surrounding shell consists of crystalline domains with varied orientations (F).

By 700 °C, polyhedral divots emerge at the center (Figure 4D), consistent with features observed during low-kV STEM and stepwise annealing experiments. Despite these morphological changes, atomic-resolution imaging reveals no significant deviations in lattice spacings, and the FFT maintains a well-defined hexagonal pattern. These results indicate that the divots correspond to a topographical change rather than a change in phase or local atomic structure. Atomic spacings in both the divot and surrounding areas remain consistent with those of TaSe_2_.

Following the complete *in situ* STEM heating cycle to 1200 °C, the sample was cooled to RT for postannealing analysis of both the core (Figure 4E) and peripheral (Figure 4F) regions. Comparing the same core site previously imaged at 700 °C (Figure 4D) and post-1200 °C (Figure 4E), we see that the polyhedral divot remains and has expanded slightly toward the left of the image. The most notable difference, however, lies in the crystal orientation. Whereas the earlier image exhibits a hexagonal arrangement consistent with the [001] direction of TaSe_2_, the postanneal image reveals an apparent tetragonal symmetry, with measured atomic spacings of 1.8 Å and 3.0 Å. These values correspond well to Ta–Ta distances in TaSe_2_ along the <101> directions, suggesting a reorientation of crystallites within the core while maintaining the material’s overall lattice structure.

A similar evolution occurs in the shell region (Figure 4F), where multiple crystalline domains appear in a range of orientations while all measured atomic spacings remain consistent with known values for TaSe_2_. These observations indicate that the entire core–shell structure has transformed into TaSe_2_ by the end of the heating cycle. FFT analysis of Figure 4F (Figure S13) further supports this conclusion: whether taken from the core, shell, or the core–shell interface, all regions display the same hexagonal pattern characteristic of TaSe_2_. Importantly, no evidence of Ta-self-intercalated TaSe_2_ was detected following this heating cycle. This absence suggests that the formation of the intercalated phase may require prolonged exposure to high temperatures.

### Proposed Transformation Pathway

The transformation pathway presented in Figure 5 takes into account all experimental and computational results obtained in this study as it captures both the chemical transformations and the multiscale morphological changes leading to core–shell architectures. This process is initiated and sustained by Se loss, which is enhanced by the high vacuum environment within the electron microscope. Chalcogen depletion is commonly observed for MX_3_-type materials and seen both under vacuum and in the presence of a carrier gas.^22,34–37,50,51^ In this system, the conversion of TaSe_3_ to TaSe_2_ involving the outermost nanoribbon material begins by 400 °C, as also previously reported by Toshima and Tanda.^35^ This temperature is higher than the 300 °C onset of weight loss seen in bulk TaSe_3_ by TGA (Figure S1F), indicating that Se loss is suppressed at the nanoscale, possibly due to confinement or surface effects. Importantly, the conversion of TaSe_3_ to TaSe_2_ produces an interfacial region with Se vacancies and associated dangling bonds, which may stabilize newly formed TaSe_2_ through interactions with neighboring TaSe_3_ chains and thereby help direct the reaction. As this this reaction front progresses, the exterior transforms into a tubular shell templated by the original nanoribbon. Upon reaching a critical threshold, the increasing lattice mismatch between TaSe_3_ and TaSe_2_ causes the newly formed TaSe_2_ layers to delaminate from the core. This separation likely occurs through shear cleavage at vdW interfaces between TaSe_2_ planes.

**Figure 5:**
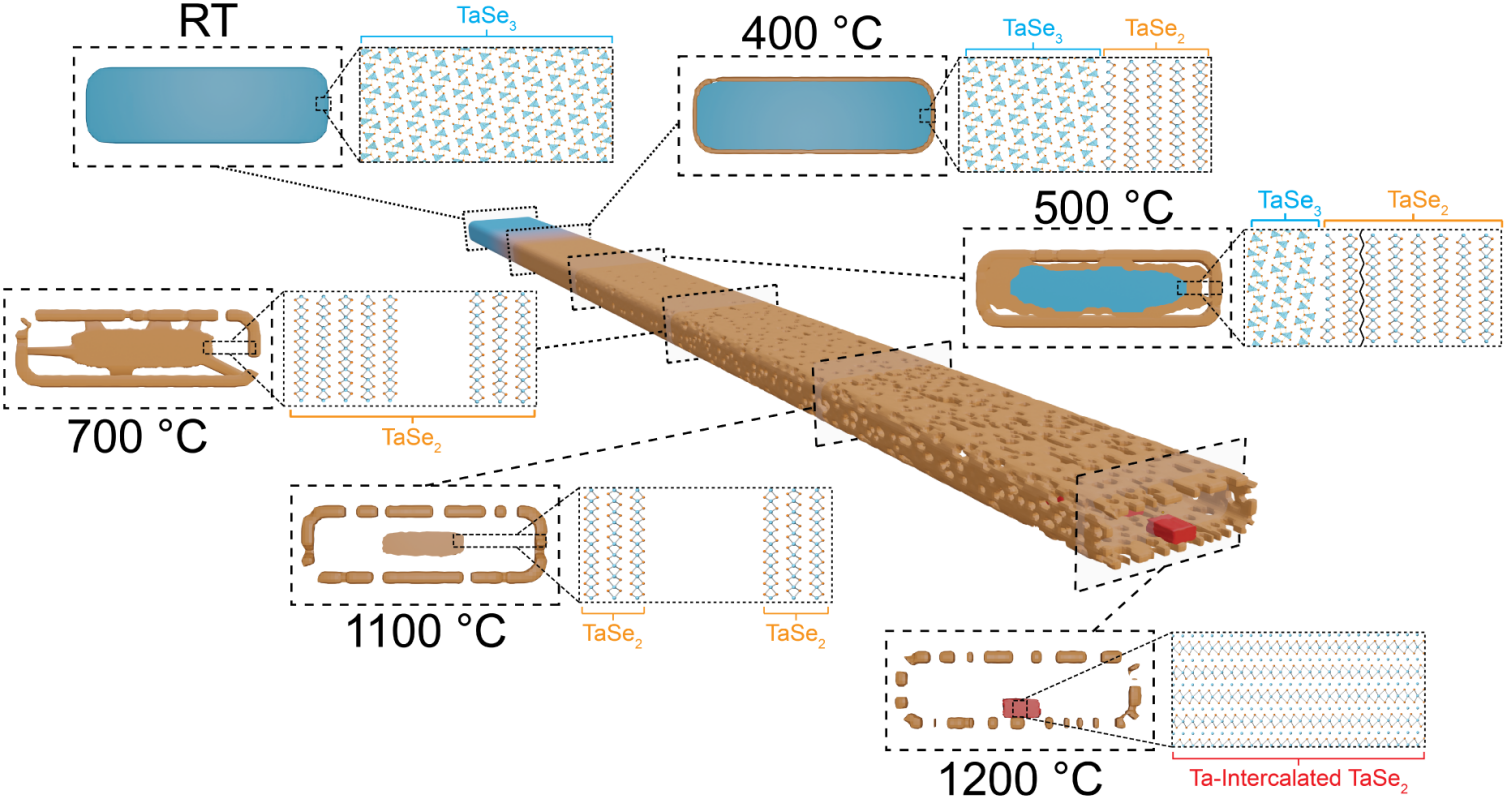
Schematic illustrating the thermally induced transformation of TaSe_3_ nanoribbons from RT to 1200 °C, with cross-sectional views highlighting key events along this pathway. RT: Initial TaSe_3_ nanoribbon cross-section shows the bilayer crystal structure viewed down the chain axis. 400 °C: Se loss from the outer layers of TaSe_3_ produces a shell of TaSe_2_. 500 °C: Cleavage of thicker TaSe_2_ from interior TaSe_3_ initiates the formation of a discrete shell nanostructure separated by void space from the core. 700 °C: All TaSe_3_ has converted into TaSe_2_, leading to increased porosity and expanded void spaces. 1100 °C: The TaSe_2_ shell has become a tubular structure with a centralized, dense core. 1200 °C: The core transforms into discrete Ta-self-intercalated TaSe_2_ nanoparticles. Continued heating at 1200 °C for >3 h ultimately causes all material to convert into elemental Ta (Figure S9).

Continued Se loss results in proliferation of void space between the core and shell, as illustrated for the 500 °C step. Imaging shows that this transformation is spatially heterogeneous as material is lost unevenly around the nanoribbon perimeter. This nonuniform breakdown gives rise to branching features and the polyhedral incursions observed in both low-kV and atomic-resolution images. Dark-field images further confirm that the core gradually adopts the hexagonal structure of TaSe_2_ and contrast differences suggest the core material is denser than that of the shell. This difference likely reflects the presence of disordered or loosely bound TaSe_2_ layers in the shell. By 700 °C, the conversion to TaSe_2_ is nearly complete, with the product retaining the outer dimensions of the starting TaSe_3_ nanoribbon despite significant internal restructuring.

At 1100 °C, the core becomes completely centralized, without the presence of branches of material extending outward to the shell seen at lower temperatures. Further heating to 1200 °C leads to the formation of discrete core nanoparticles. This final transformation involves Ta migration and self-intercalation into the vdW gaps of TaSe_2_ driven by the increasing relative concentration of Ta as Se loss progresses. The unit cell expands with Ta intercalation to adopt the experimentally observed lattice. This pathway to a self-intercalated phase is quite different from conventional routes utilizing vapor growth (MBE, CVD, CVT).^42–44^

Strikingly, the core–shell nanostructure appears to act as an *in situ*-generated nanoreactor: the TaSe_2_ shell encapsulates and stabilize intercalated TaSe_2_ core nanoparticles, which otherwise are not observed. We speculate that this shell also modulates the kinetics of Se loss to favor the formation of the core particles at prolonged high temperatures. Recent reports have described other core–shell nanoreactors that stabilize new phases, modulate reactivity, or modify the properties of encapsulated materials.^52,53^ However, most of these studies rely upon nanocrystal growth within nanotubes to achieve heterogeneous core–shell assemblies. In comparison, what we describe here is the *in situ* formation of a tantalum–selenium core–shell architecture; this product is “emergent” in the sense of developing spontaneously upon heating through the interplay of atomic mobility and confinement effects.

Comparing this mechanism to our previous study on NbS_3_–IV highlights the surprising system-specific thermal breakdown behavior of MX_3_-type materials.^22^ NbS_3_–IV converts to nanostructured NbS_2_ through a topotactic transformation, resulting in grain formation parallel to the nanoribbon axis and accompanied by a marked decrease in nanoribbon width. In contrast, TaSe_3_ converts to TaSe_2_ in a layered fashion, causing randomly oriented grains of TaSe_2_ to form a robust shell surrounding a core of TaSe_2_ with little impact on its overall width. Only TaSe_3_ gives rise to a metal dichalcogenide core that further transforms into a self-intercalated metal dichalcogenide phase. These findings demonstrate the need to carefully examine MX_3_ reaction pathways on a case-by-case basis to uncover unique chemistries.

## Conclusions

This study provides a comprehensive view of the high-temperature structural and chemical evolution of TaSe_3_ nanoribbons, enabled by low-kV and aberration-corrected *in situ* STEM, EELS, and DFT calculations. These experiments revealed a multistep transformation that culminates in unique, multiphase 0D–1D core–shell architectures. This work allowed us to formulate a detailed mechanism with several unexpected features. The first is the formation of a porous, tubular TaSe_2_ shell at temperatures of 400–1000 °C. The second is the formation of a Ta-self-intercalated phase within the TaSe_2_ tubes at 1000–1200 °C, a process that appears to be enabled by nanoscale spatial confinement. These findings suggest that core–shell architectures, like those seen in this system, can act as “nanoreactors” in which the local environment can promote otherwise inaccessible phase behavior and chemistry.

The broader implications of this work are significant. It introduces a novel route to fabricating complex, mixed-dimensional architectures in a technologically relevant transition metal chalcogenide system. The resulting structures may offer enhanced or tunable functionality, such as catalytic activity or charge transport behavior, although further work is needed to develop *ex situ* preparative routes.^54,55^ Thus, despite being traditionally viewed as “decomposition” reactions, thermally driven transformations like the ones described here can be reframed as deliberate design principles or synthetic strategies. In this way, this study highlights new opportunities for accessing intricate architectures from relatively simple precursors and can inspire synthetic innovation.

Beyond elucidating fundamental reaction pathways, this work has practical value for evaluating the thermal stability of low-dimensional metal chalcogenides. As TaSe_3_ and related MX_3_ compounds are increasingly considered for nanoelectronics, optoelectronics, and quantum technology applications, it becomes important to understand their behavior under processing- and operation-relevant conditions.

These findings also underscore the power of real-time, high-resolution imaging in advancing our understanding of solid-state nanochemistry. In particular, this study demonstrates the unique advantages of *in situ* electron microscopy to directly capture the structural and compositional evolution of 1D nanomaterials, thereby enabling mechanistic insights into complex transformation pathways across atomic-to-nano length scales.

## Experimental Methods

### Preparation of TaSe_3_

TaSe_3_ crystals were synthesized by a melt-flux reaction with a Ta:Se molar ratio of 1:6 adapted from Xia et al.^31^ The excess Se was used as a reactive self-flux. Elemental powders of Ta (0.2766 g; Strem Chemicals, Inc.; 99.98%) and Se (0.7232 g; Strem Chemicals, Inc.; 99.99%) were ground and thoroughly mixed using a pestle and mortar. The powder was deposited into a fused quartz ampule with volume ∼33 cm^3^. The ampule and its contents were backfilled with argon and evacuated 3 times on a Schlenk line while keeping the ampule submerged in a cooling bath of dry ice and acetonitrile. The ampule was placed into a Lindberg/Blue M box furnace and heated up to 750 °C over 9 h. The sample was held at 750 °C for 20 h before slowly cooling to 450 °C at a rate of 1.5 °C h^–1^. Upon reaching 450 °C, the ampule was allowed to cool to RT naturally. The product consisted of a dense pellet with growths of wispy, metallic fibers on top. Bulk TaSe_3_ was collected by manually separating the fibers from the pellet.

### Bulk TaSe_3_ Characterization

TaSe_3_ was characterized by powder X-ray diffraction using a Bruker D8 Advance diffractometer using a ^60^Co–Kα source (λ = 1.78890 Å) under ambient conditions. The diffraction pattern was collected at 35 kV and 40 mA between 5° and 80° 2θ with a scan speed of 0.1 s/step and step increment of 0.01°. The obtained pattern was matched to TaSe_3_ (ICDD PDF: 04-007-1143) using the PDF4+ database.

Bulk TaSe_3_ crystals were mounted to a SEM stub and mechanically exfoliated using Scotch tape. SEM images were acquired using a Thermo Fisher Scientific Teneo FE-SEM with an accelerating voltage of 30 kV. Energy dispersive X-ray spectroscopy data were collected using an Oxford X-Max^N^ 150 large area silicon drift detector.

TGA of bulk TaSe_3_ crystals was performed using a PerkinElmer TGA 8000 thermogravimetric analyzer. Data collection was conducted under N_2_ gas flow while the temperature was ramped at a rate of 10 °C min^–1^ from 30 to 1000 °C.

### Exfoliation of Bulk TaSe_3_

Liquid-assisted exfoliation yielded sufficiently thin nanoribbons for electron microscopy studies. ∼3 mg of TaSe_3_ was placed into a centrifuge tube along with 10 mL of degassed 100% ethanol. A probe sonicator micro tip (Misonix S-4000 Sonicator with Model CL5 converter) was immersed into the liquid and operated for 3 h with an amplitude of 30. An ice–water bath was used and periodically replaced to ensure the ethanol did not evaporate during the sonication process. The resultant dispersion appeared cloudy and dark gray in color.

### Sample Loading onto MEMS Chips

Dispersed TaSe_3_ nanoribbons were brushed onto the sample heating wells of either a Norcada MEMS chip (for low kV STEM analysis) or Protochips MEMS chip (for atomic resolution STEM analysis). A Kimwipe tissue laden with the exfoliated nanoribbon dispersion was used as the brush.

### Low kV *In Situ* STEM Thermolysis

Norcada MEMS chip with TaSe_3_ nanoribbons within the heating area was inserted into a Hitachi Blaze single tilt holder. Subsequently, the holder was inserted into a Hitachi SU9000EA STEM and images were collected using secondary electron, bright field, and high angle annular dark field detectors simultaneously. Images were collected using an accelerating voltage of 30 kV and current of 10 μA. Using a heat ramp of 5 °C/s, images were collected at 100 °C temperature steps between RT and 1000 °C. EDS was taken with an Oxford Ultim Extreme attached to the SU9000EA STEM operating at 30 kV.

### Atomic Resolution *In Situ* STEM/EELS Thermolysis

Protochips MEMS chips with TaSe_3_ nanoribbons deposited into the heating area were used inside a Nion UltraSTEM 100 operated at 100 kV for both the *in situ* annealing and temperature step experiments. The experiments mirrored similar conditions to the low kV STEM experiment with the same heat ramp of 5 °C/s for both experiments and 100 °C increments for the temperature step experiment. The maximum temperature at which images were collected was 1200 °C. Images were not collected immediately upon reaching target temperature due to the necessity of thermally equilibrating the sample stage to mitigate drift.

### Data Processing and Measurements

STEM images were processed using ImageJ to optimize the brightness and contrast settings. All STEM image measurements were obtained using ImageJ. Periodic-plus-smooth image decomposition was completed using Correlescence v.0.0.6 plugin for ImageJ (https://github.com/ekatrukha/Correlescence). CrystalMaker 11 was used to measure simulated crystal structures. SingleCrystal 5.0.0 was used for electron diffraction pattern simulation.

### First-Principles Calculations

First-principles calculations based on density functional theory (DFT) were performed using the Vienna Ab Initio Simulation Package (VASP) with a plane-wave basis set, in conjunction with the generalized gradient approximation of Perdew, Burke, and Ernzerhof and the projector augmented wave method.^56–60^ Spin-polarized calculations were performed, with 520 eV cutoff energy for the plane wave basis set, 10^–6^ eV energy convergence criteria and 21 × 21 × 8 k-point mesh. Structural relaxations were performed at 0 K, allowing full relaxation of lattice parameters, atomic positions, and cell symmetry until the maximum force on each atom was <0.001 eV Å^–1^. Enthalpies of formation were obtained from the total energies of the optimized structures, with reference to the energies of elemental Ta and Se in their most stable phases.

## Supplementary Material



## Notes

TaSe_3_ crystal structure and material characterization data; full temperature range for low kV *in situ* STEM; plot of nanoribbon width by detector mode vs temperature; low kV *in situ* STEM images of shell pore expansion; high- and low-defocus electron micrographs; shell, core–shell interface, and core delineation; low-magnification view of transformed core particles; nearest Ta–Ta spacings; plot of nanoribbon width vs anneal step; SEM-EDS of sample after extended heating; core particle comparison to TaO; convex hull for Ta–Se binary system; core particle measured atomic distances; post-1200 °C core–shell FFT measurements (PDF)
