# Supplementary material for: Thermally Driven Formation of Multiphase, Mixed-Dimensional Architectures from TaSe3 Nanoribbons
Source: ACS Nano. 2025 Oct 20;19(43):38028–39. doi: 10.1021/acsnano.5c13312 (PMC12593384; doi:10.1021/acsnano.5c13312)
Supplement: Supplementary file 1 [file nn5c13312_si_001.pdf]

Supporting Information

Thermally-Driven Formation of Multiphase, Mixed-Dimensional Architectures from $TaSe_3$ Nanoribbons

Casey F. Rowe[1], Eric V. Formo[2*], Jordan A. Hachtel[3], Tina T. Salguero[1*]

1) Department of Chemistry, University of Georgia, Athens, GA USA
2) Georgia Electron Microscopy, University of Georgia, Athens, GA USA
3) Center for Nanophase Materials Sciences, Oak Ridge National Laboratory, Oak Ridge, TN USA

* Authors to whom correspondence should be addressed
eformo@uga.edu
salguero@uga.edu

SI Contents:

Figure S1	$TaSe_3$ crystal structure and bulk sample characterization (PXRD, SEM, EDS, and TGA)
Figure S2	Full temperature range (100 °C increments) for low kV *in situ* STEM
Figure S3	Plot of nanoribbon width according to detector mode vs. temperature
Figure S4	Low kV *in Situ* STEM images showing development of shell porosity
Figure S5	High and low defocus electron micrographs of periphery and interior regions
Figure S6	Shell, core-shell interface, and core delineation and key transformation sites
Figure S7	Low magnification view of transformed core particles
Table S1	Nearest Ta-Ta spacings
Figure S8	Plot of nanoribbon width vs. anneal step
Figure S9	SEM-EDS of sample after extended heat treatment
Figure S10	Core particle atomic distance measurement comparison to TaO
Figure S11	Convex hull for Ta–Se binary system
Figure S12	Measured atomic distances for core particles
Figure S13	FFTs from distinct regions of the post-1200 °C core-shell architecture

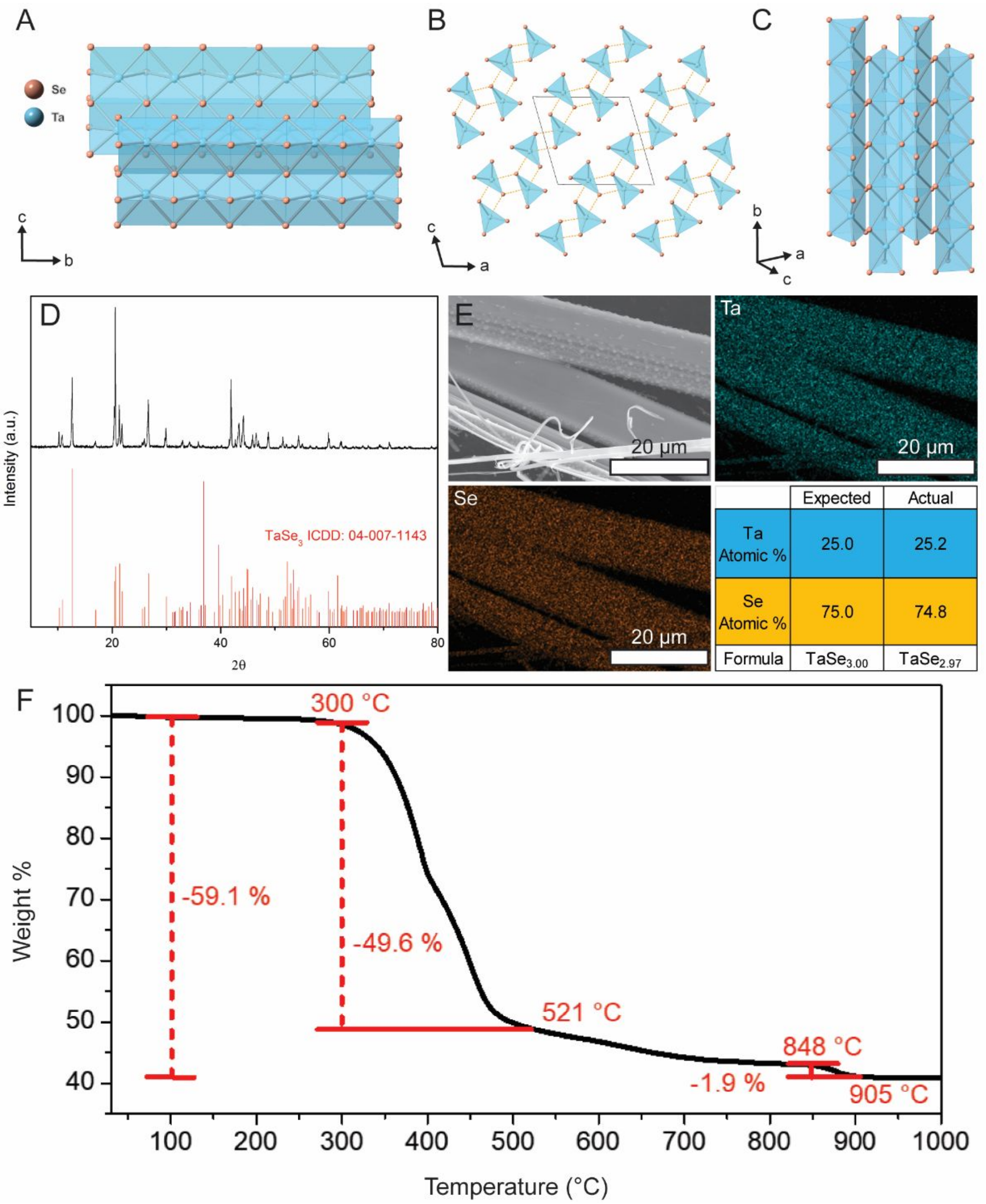

Figure S1. Crystal structure of quasi-1D $TaSe_3$: down the *a*-axis (A), down the chain-axis (*b*-axis) with a view of the van der Waals gaps between chain bilayers (B) (dashed yellow lines indicate relatively weak dative bonds between chains and the black box indicates the unit cell), and a clearer view of the stacked trigonal prismatic structure of $TaSe_3$ chains (C). Characterization of as-synthesized bulk $TaSe_3$ crystals: PXRD and comparison with ICDD reference pattern 04-007-1143 (D), SEM of bulk $TaSe_3$ crystals with EDS maps for Ta and Se and measured atomic percentages (E), and TGA of bulk $TaSe_3$ conducted under N_2 gas flow (F).

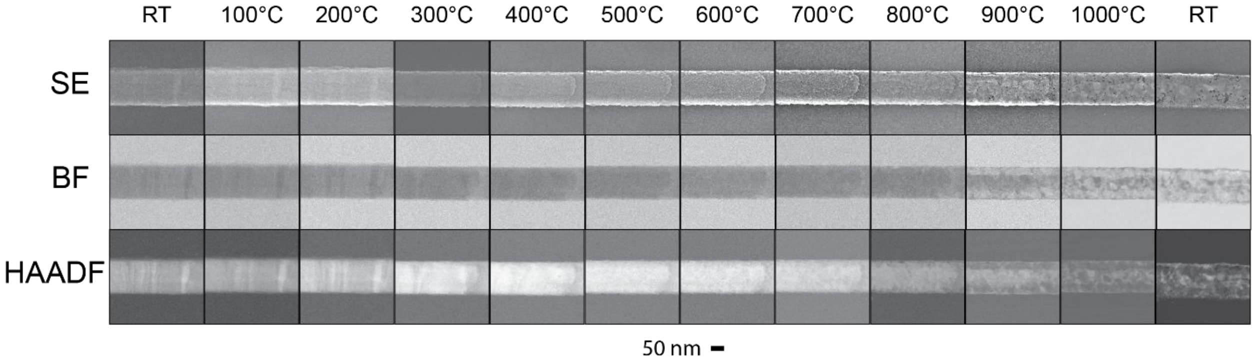

Figure S2. *In situ* thermolysis STEM temperature study of $TaSe_3$ nanoribbon in 100 °C increments using a 30kV Hitachi SU9000EA microscope with secondary electron (SE), bright field (BF), and high angle annular dark field (HAADF) detectors. All images show the same nanoribbon. However, some image locations are shifted to account for contamination and beam damage issues.

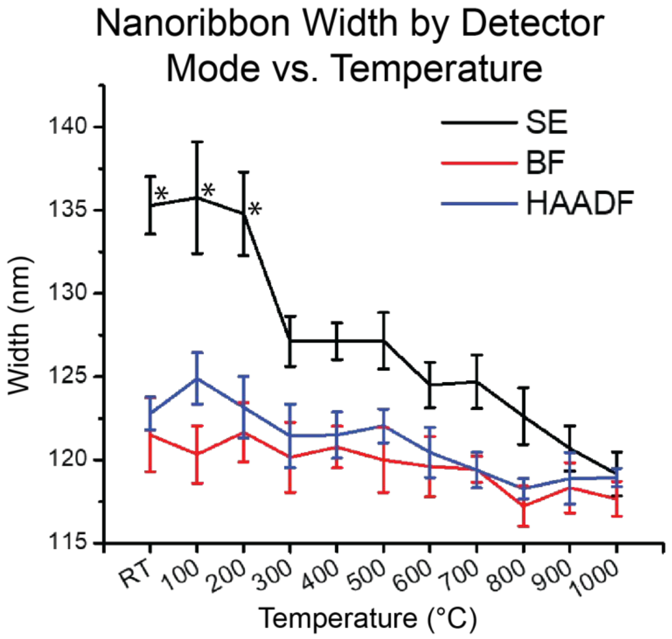

Figure S3. Nanoribbon widths, as measured by SE, BF, and HAADF detectors on the SU9000EA microscope, as a function of temperature. Asterisks indicate the presence of a visible organic overlayer.

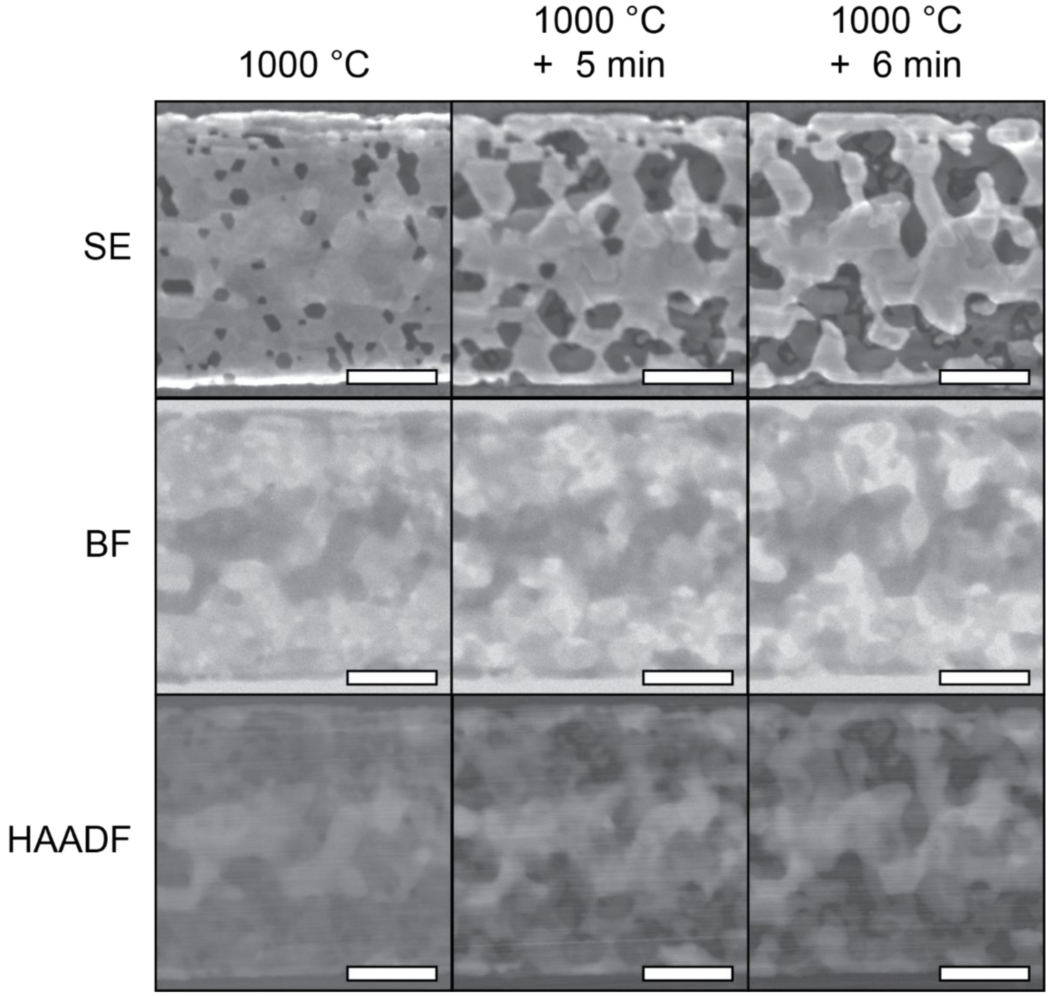

Figure S4. SE, BF, and HAADF images of the transforming nanoribbon at 1000 °C that highlight the rapid increase of $TaSe_2$ shell porosity. Scale bars = 50 nm.

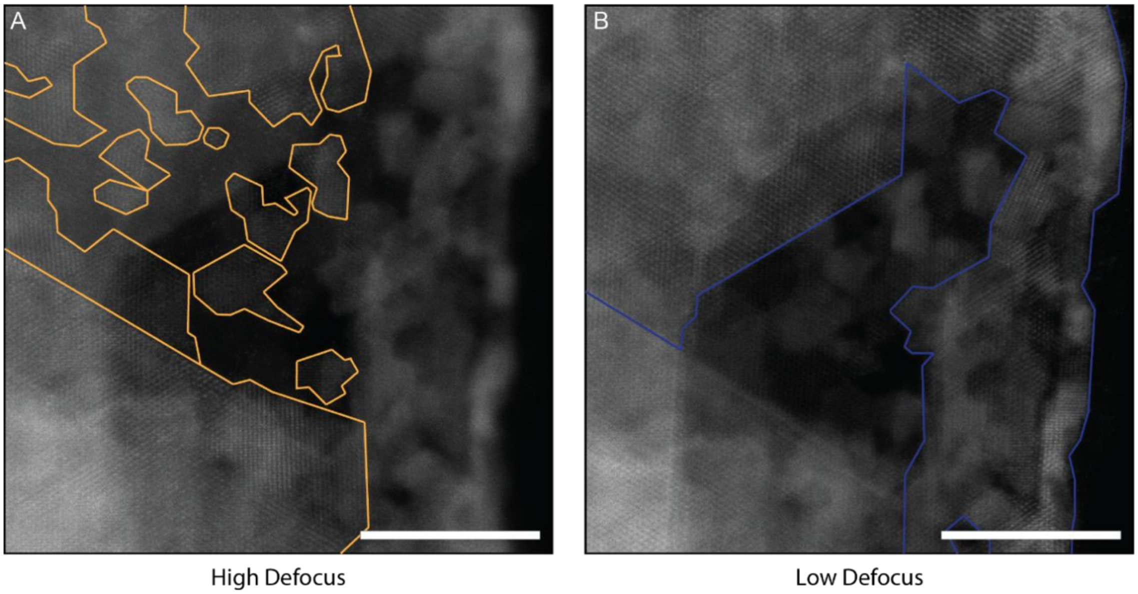

Figure S5. Product of annealing $TaSe_3$ nanoribbon at 1200 °C, imaged using a Nion UltraSTEM 100 microscope, showing views of the periphery and interior regions at high and low defocus. The comparison of interior features at high defocus (A) vs. periphery features at low defocus (B) demonstrates that the product retains 3D features. Scale bars = 10 nm.

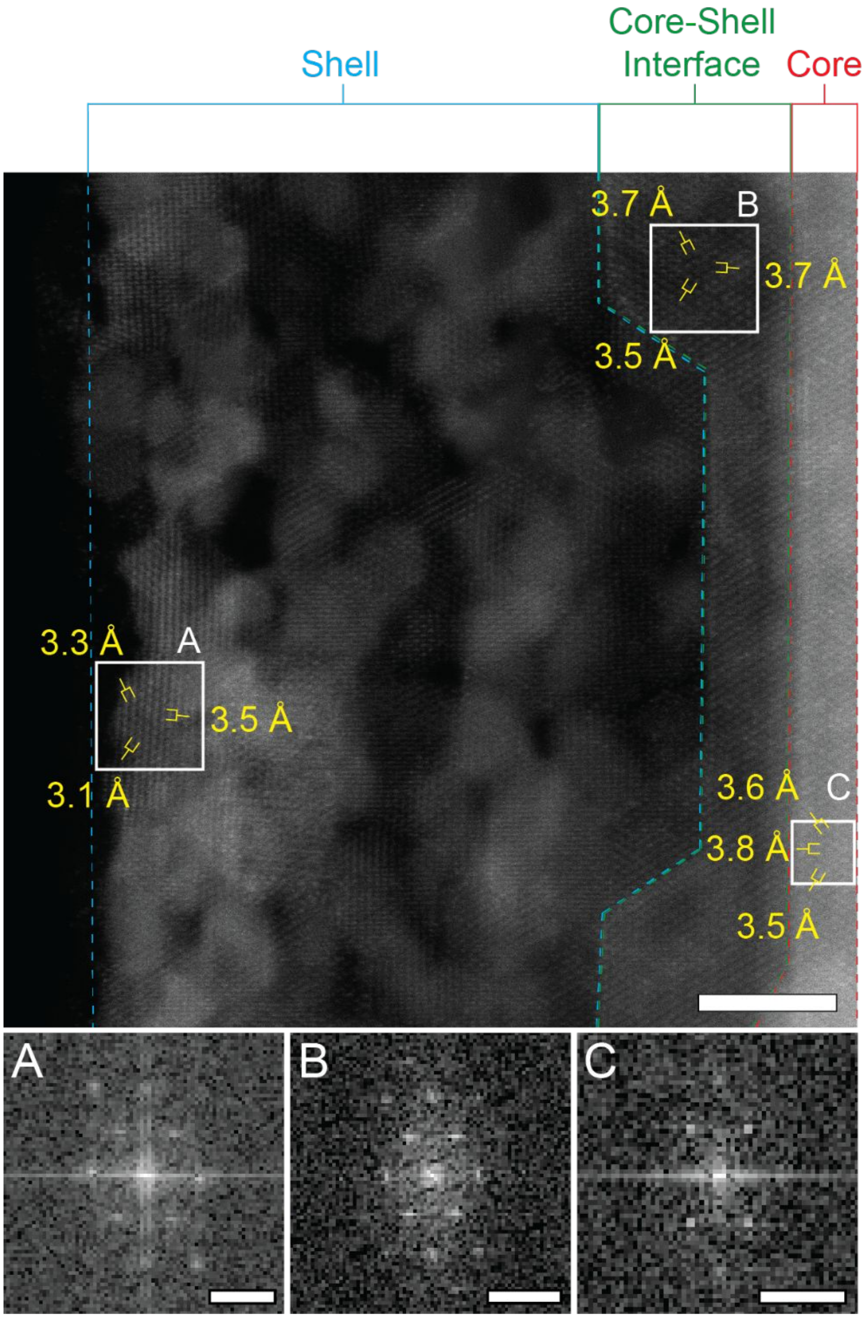

Figure S6. Key transformations sites within the 1200 °C-annealed $TaSe_3$ nanoribbon: shell (A), core-shell interface (B), and core (C). FFT patterns correspond to the boxed areas. Scale bar = 5 nm. FFT scale bars = 0.5 $Å^{-1}$.

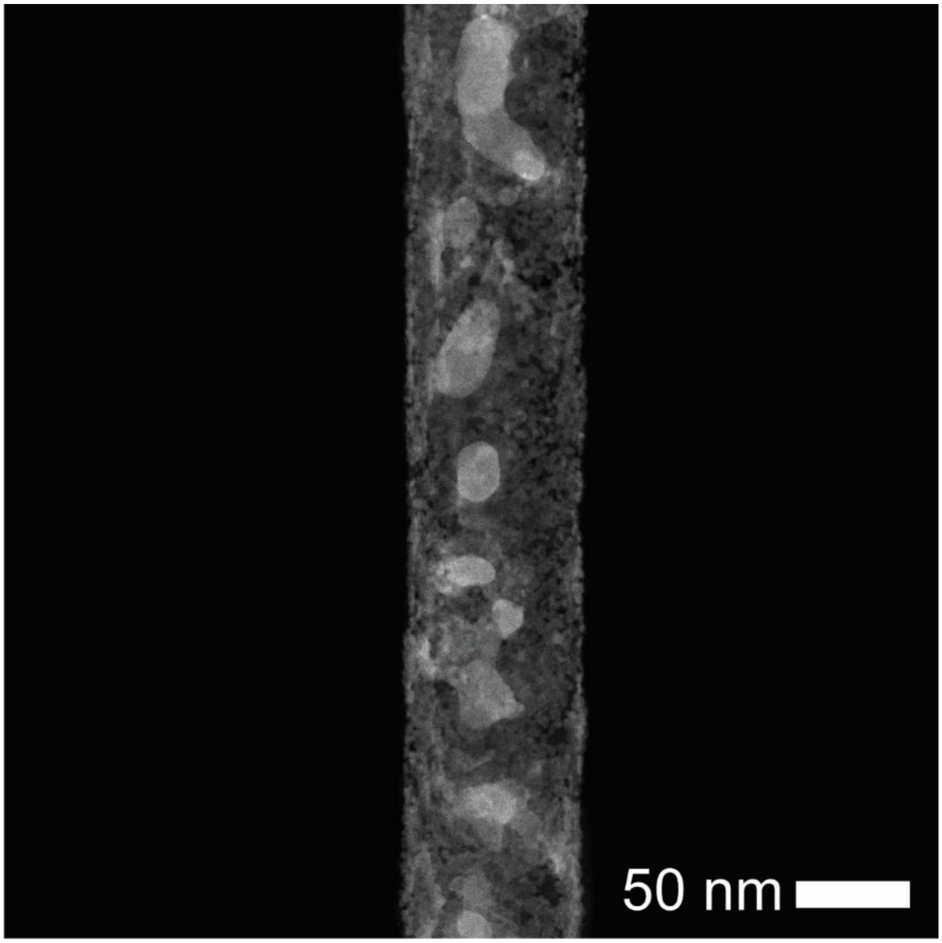

Figure S7. Image of a transformed $TaSe_3$ nanoribbon after 1200 °C heating that clearly shows the discrete core particles.

Table S1. Table of nearest Ta – Ta spacings for binary Ta–Se phases and Ta according to measured screen distance in specified directions.

		Nearest Ta - Ta Screen Distance (Å)						
**ICDD PDF #**	**Phase**	**[100]**	**[010]**	**[001]**	**[110]**	**[101]**	**[011]**	**[111]**
01-073-1798	1T-$TaSe_2$	3.0	3.0	3.5	3.0	3.0	3.0	3.0
01-073-1799	2H-$TaSe_2$	3.0	3.0	3.4	3.0	1.7	1.7	1.7
01-073-1800	3R-$TaSe_2$	3.0	3.0	3.5	3.0	1.1	1.1	1.1
01-073-1802	4H(b)-$TaSe_2$	3.0	3.0	3.5	3.0	0.9	0.9	0.9
01-073-1803	6R-$TaSe_2$	3.0	3.0	3.5	3.0	1.2	1.2	1.2
00-004-0788	α-Ta	2.3	2.3	2.3	1.7	1.7	1.7	2.7
00-021-0603	Ta_2Se_3	1.7	2.4	1.3	2.7	1.0	1.7	0.8
01-079-0779	Ta_2Se	2.3	2.3	2.4	1.5	1.0	1.0	1.4

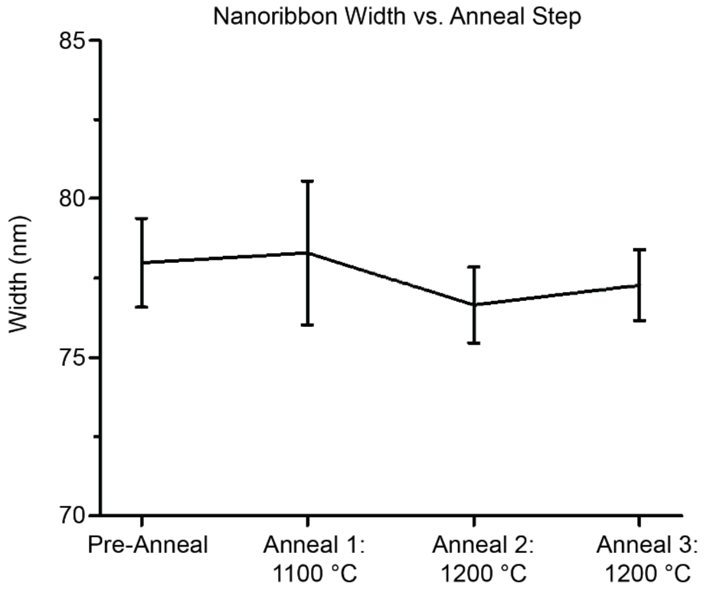

Figure S8. Plot of measured nanoribbon widths after each anneal step (Nion UltraSTEM 100).

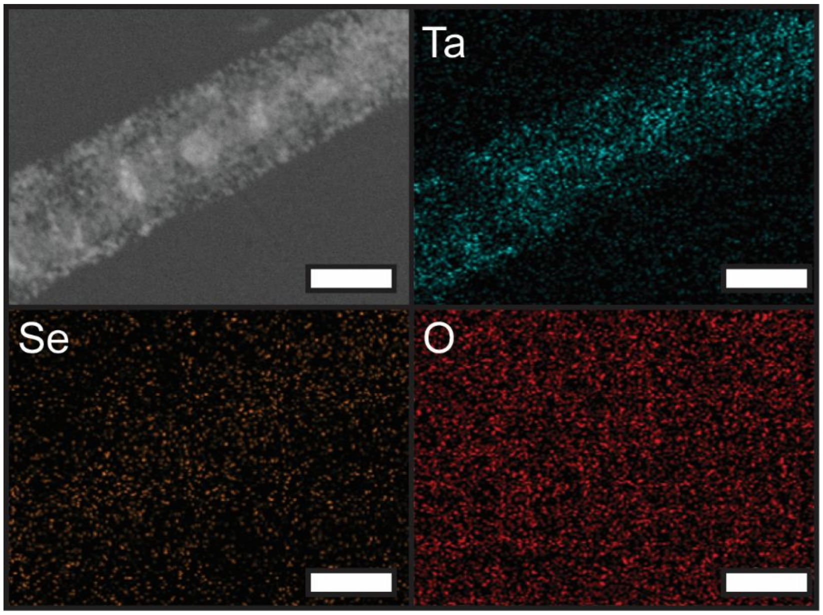

Figure S9. SEM-EDS analysis of the transformed core-shell nanostructure after heating at 1000 °C for an extended time (>3 h). Scale bars = 100 nm.

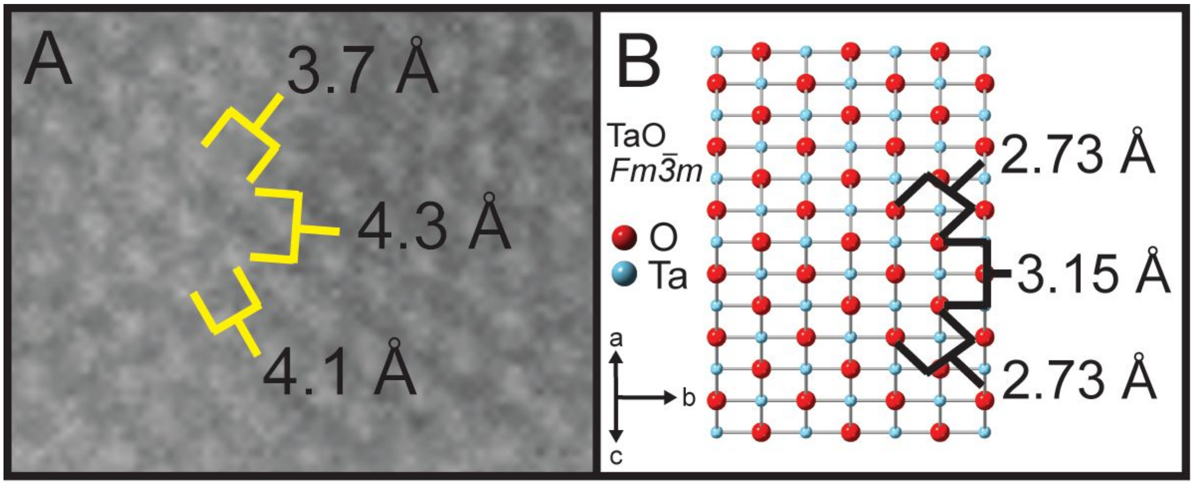

Figure S10. Comparison of the observed atomic spacings from a representative core particle (A) with the calculated spacings for cubic TaO (B).

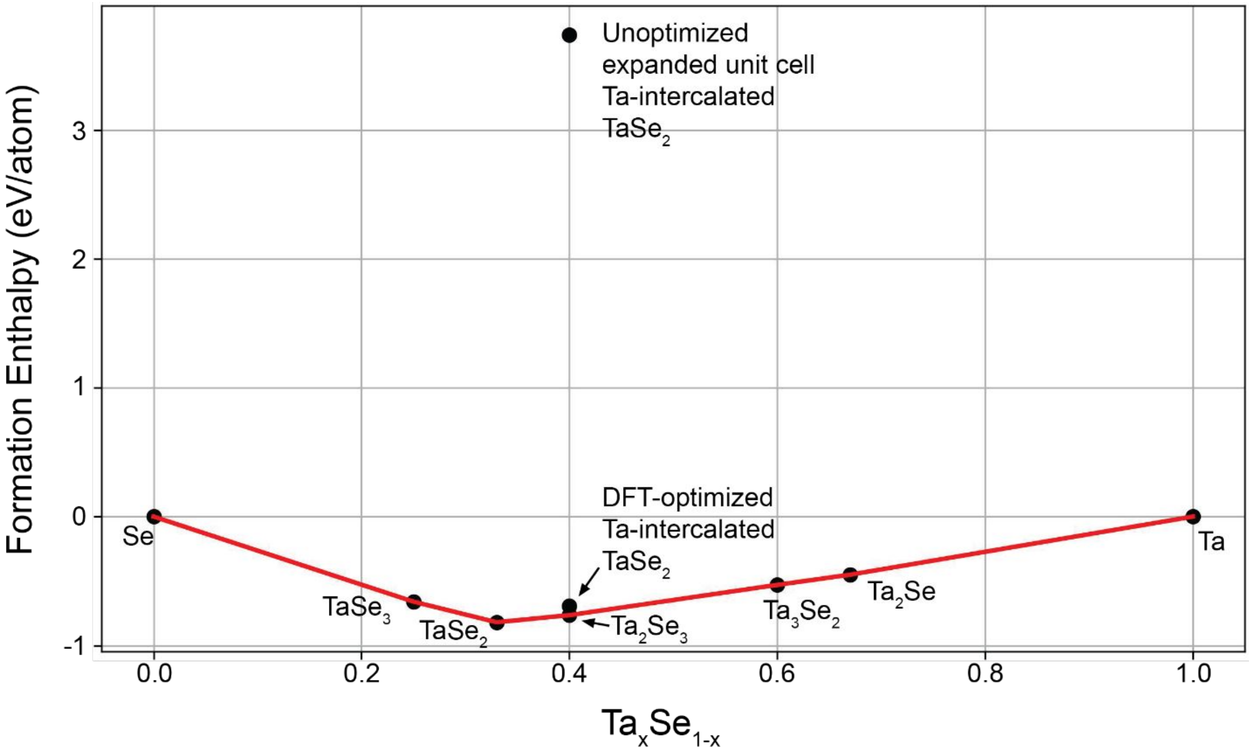

Figure S11. Convex hull for Ta–Se binary system including both DFT-optimized and unoptimized Ta-intercalated $TaSe_2$ phases calculated at 0 K.

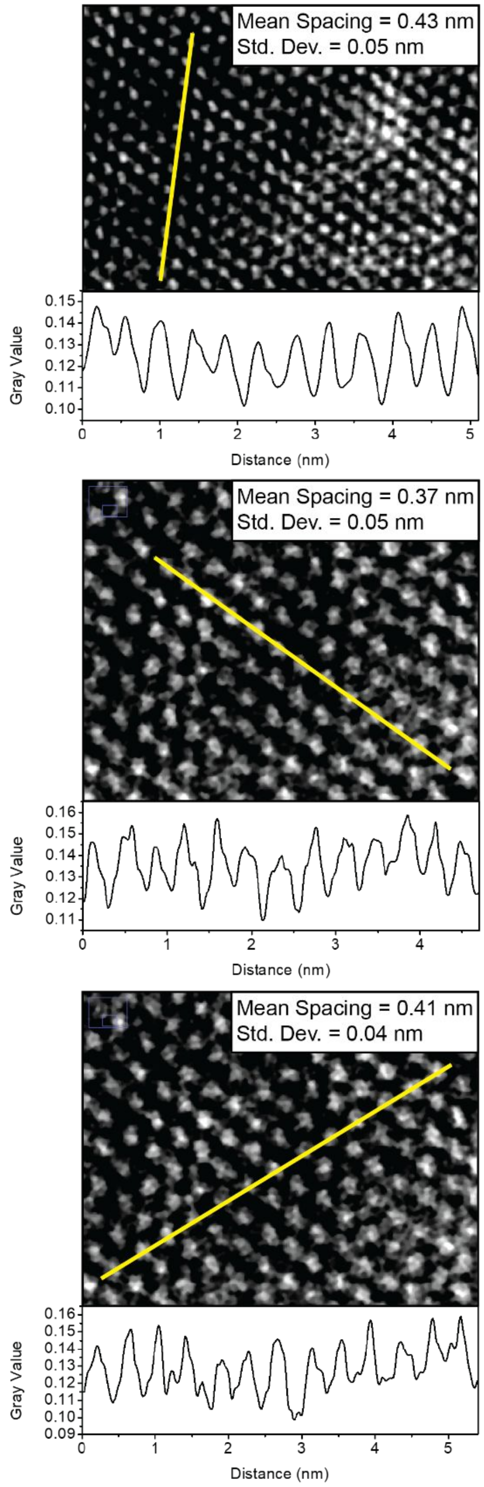

Figure S12. Gray value line profiles for STEM images of core particle showing measured distances between atoms.

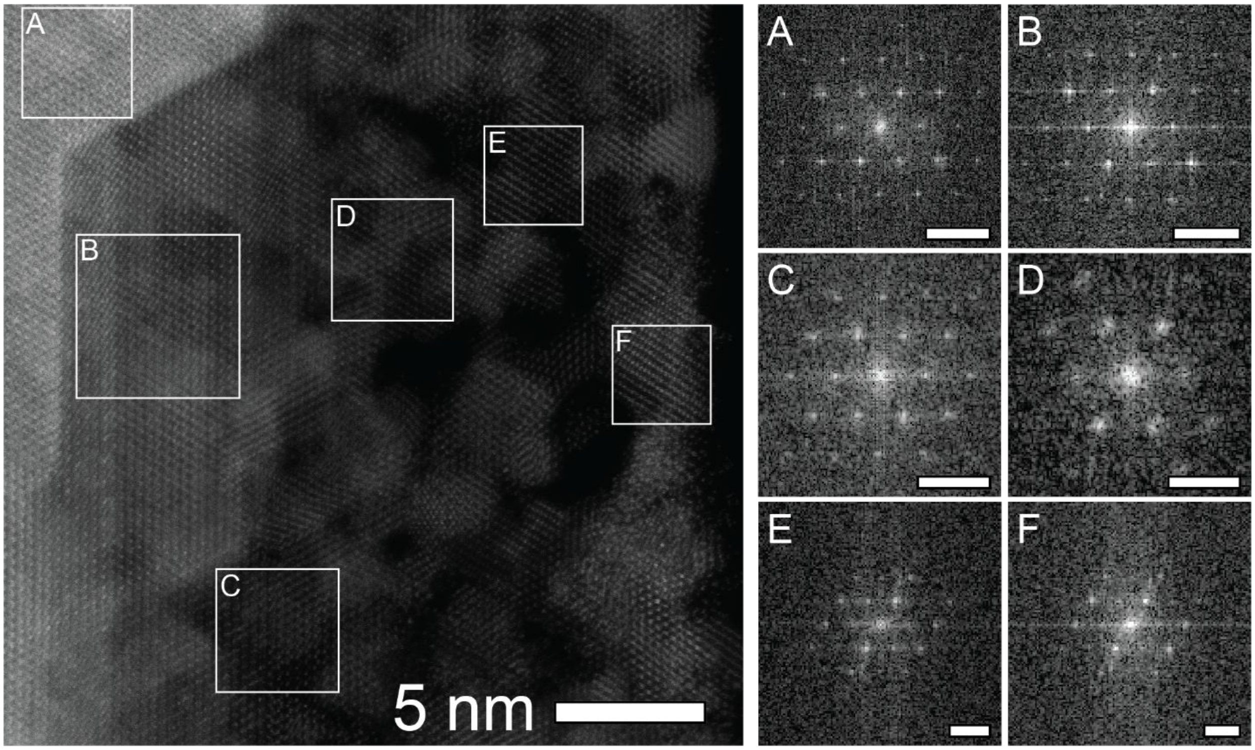

Figure S13. FFTs of core (A), core-shell interface (B), and shell (C-F) areas after sample heating to 1200 °C. FFT inset scale bars = 0.5 $Å^{-1}$.
